# Association between ß2-adrenergic receptor gene polymorphisms and adverse events of ritodrine in the treatment of preterm labor: a prospective observational study

**DOI:** 10.1186/s12863-017-0565-8

**Published:** 2017-11-13

**Authors:** Jee Eun Chung, Soo An Choi, Han Sung Hwang, Jin Young Park, Kyung Eun Lee, Jeong Yee, Young Ju Kim, Hye Sun Gwak

**Affiliations:** 1 School of Pharmacy, Sungkyunkwan University, Suwon-si, Gyeonggi-do 16419 Korea; 2 College of Pharmacy, Korea University, Sejong-Si, 30019 Korea; 3 Department of Obstetrics and Gynecology, Konkuk University Medical Center, Konkuk University School of Medicine, Seoul, 05030 Korea; 4 College of Pharmacy and Division of Life & Pharmaceutical Sciences, Ewha Womans University, 52 Ewhayeodae-gil, Seodaemun-gu, Seoul, 03760 Republic of Korea; 5 College of Pharmacy, Chungbuk National University, Cheonju, Chungbuk 28644 Korea; 6 Department of Obstetrics and Gynecology, Ewha Womans University School of Medicine, 1071 Anyangcheon-ro, Yangcheon-gu, Seoul, 07985 Republic of Korea

**Keywords:** Ritodrine, β2-agonist, β2-adrenergic receptor, Tocolysis, Adverse drug events, Gene polymorphism

## Abstract

**Background:**

Ritodrine, a tocolytic β2-agonist, has been used extensively in Europe and Asia despite its safety concerns. This study was designed to identify associations between β2-adrenergic receptor (*ADRB2*) polymorphisms and adverse drug events (ADEs) in patients with preterm labor treated with ritodrine.

**Results:**

This follow-up study was prospectively conducted at Ewha Womans University Mokdong Hospital in Korea. Five single nucleotide polymorphisms (SNPs) of the *ADRB2* gene (rs1042713, rs1042714, rs1042717, rs1042718, and rs1042719) were analyzed in 186 pregnant women with preterm labor. Patients with the AA genotype of rs1042717 had significantly lower incidence of ADEs compared to those with the G allele (*p* = 0.009). In multivariate analysis, one of the predictors of ADEs was the maximum infusion rate of ritodrine (AOR 4.47, 95% CI 1.31–15.25). Rs1042719 was also a significant factor for ritodrine-induced ADEs. The CC genotype carriers had 78% decreased risk of ADEs compared to those with other genotypes.

**Conclusions:**

This study demonstrates that ADEs induced by ritodrine are associated with *ADRB2* gene polymorphisms, as well as the infusion rate of ritodrine in pregnant women with preterm labor.

## Background

Preterm birth, defined as birth that occurs before the completion of 37 weeks of pregnancy, is known as the primary cause of perinatal mortality and morbidity [1]. In addition, it is also the second most frequent cause of infant mortality, ranking only behind birth defects [2]. The mechanisms of preterm labor are not well explained. Reasons for spontaneous preterm labor may involve mechanical and genetic factors, the inflammatory response, stress, infection, and hemorrhage [3].

Tocolytics are often used to prolong pregnancy when preterm labor occurs. One particular tocolytic that has been used extensively is the β2-agonist. The β2-adrenergic receptor (ADRB2) is predominant in the human myometrium [4]. Beta-2 agonists such as ritodrine inhibit myometrial contractions by stimulating adenylyl cyclase activity via ADRB2. In this way, cytosolic cyclic adenosine monophosphate (cAMP) is generated, which activates protein kinases and phosphorylates cytosolic and membrane proteins. Consequentially, there is reduced interaction between actin and myosin, which is the key factor of cell contraction [5].

Despite the therapeutic usefulness of β2-agonists, they have lost popularity because of safety concerns. Several studies have compared β2-agonists to other tocolytics, such as oxytocin antagonists and calcium channel blockers. There were no differences in efficacy such as delay in delivery; however, there were differences in the incidence rates of adverse drug events (ADEs), especially cardiovascular side effects [6–8]. The ADRB2 plays a pivotal role on the regulation of heart rate, contractility, vasodilation, and bronchodilation. It is also involved in smooth muscle relaxation of the myometrium. The ADEs induced by β2-agonists not only include tremors, dyspnea, headaches, and palpitations, but also adverse cardiac events, such as tachycardia, elevated systolic blood pressure, chest pain, and myocardial ischemia. Due to the ADE issues, it was withdrawn in the USA in 1998. However, it is still being used in Asian and European countries.

Various factors, including maternal age, concurrent medications and clinical factors can affect the efficacy and safety among patients treated with β2-agonists. Genetic polymorphisms have been implicated as additional factors affecting drug effectiveness and ADEs. There are several candidate genes that may explain the variation in β2-agonist effects. Most prior studies were conducted in respiratory or cardiovascular diseases [9–11].

The human *ADRB2* gene on chromosome 5 is abundantly expressed in cardiac myocytes and vascular smooth muscle cells. It has three major missense polymorphisms, which encode amino acids 16, 27, and 164 of the extracellular N-terminus of the β2-adrenoceptor [12, 13]. A previous in vivo study revealed that the genetic variants in *ADRB2* could be a predictive marker for the responsiveness of β2-agonists [13]. In our previous clinical study, we also found that the rs1042719 polymorphism had a significant effect on time to delivery in both univariate and multivariate analyses; compared to the wild-type homozygote carriers, the GC and CC carriers showed a 64% decrease in time to delivery [14].

In terms of safety, the polymorphism of Arg16Gly (rs1042713) appeared to be associated with ADEs in asthmatic patients treated with short acting β2-agonists [15]. However, no prior studies have addressed the association between genetic polymorphisms and ADEs in patients with preterm labor treated with β2-agonists. Therefore, we sought to evaluate the relationship between *ADRB2* polymorphisms and ADEs in pregnant women with preterm labor treated with ritodrine.

## Results

A total of 216 women were hospitalized for threatened preterm labor and were treated with ritodrine. Patients who had already experienced severe symptoms requiring urgent surgery (*N* = 14), a plan for the McDonald operation (*N* = 8), or underlying cardiovascular diseases (*N* = 8) were excluded. A total of 186 patients were ultimately enrolled in our study.

Table 1 displays the demographic and clinical characteristics of the study population stratified by ADE occurrence. The mean maternal age was 30.9 (±4.7) years, and the mean gestational age at the time of hospitalization was 29.6 (±3.9) weeks. Multiple gestation pregnancies were observed in 8.6% of the study population. ADEs occurred in 33 patients (17.7%). Most ADEs included tremor, palpitations, dyspnea, and tachycardia, along with one case of pulmonary edema. With the exception of the maximum infusion rate, there was no significant difference between the two groups. The infusion rate was lower in the group with no adverse events than in the group with adverse events (*P* = 0.006).

**Table 1:** Demographic and clinical characteristics of the study population

Characteristics	Total (*n* = 186)	Adverse Event Group (*n* = 33)	No Adverse Event Group (*n* = 153)	*P*-value
Age, years	30.88 ± 4.70	31.21 ± 3.45	30.81 ± 4.48	.635
Body mass index, kg/m^2^	24.50 ± 3.19	24.97 ± 2.98	24.40 ± 3.23	.354
Gestational age at start of therapy, weeks	29.60 ± 3.91	28.97 ± 4.23	29.74 ± 3.85	.308
Maximum infusion rate, mg/min	0.083 ± 0.030	0.096 ± 0.044	0.080 ± 0.026	.006
Comorbid conditions, N (%)				
Twin or multiple gestation	16 (8.6)	4 (12.1)	12 (7.2)	.491
Diabetes mellitus	13 (7.0)	3 (9.1)	10 (6.5)	.729
Uterine infection	15 (8.1)	1 (3.0)	14 (9.2)	.475

Data are expressed as means ± SDs, unless otherwise specified

The effects of 5 SNPs of the *ADRB2* gene on ADEs were evaluated (Table 2). Minor allele frequencies (MAFs) in our study population ranged between 10 and 47%. All of the allele frequencies were consistent with the Hardy-Weinberg equilibrium. The two polymorphisms (rs1042718 and rs1042717) were in linkage disequilibrium (LD) in this study population (r^2^ = 0.98). In univariate analysis, rs1042717 (G > A), rs1042718 (C > A), and rs1042719 (G > C) were significantly associated with ADEs. In variant homozygous carriers of rs1042717 (as well as rs1042718), ADEs were not identified. In contrast, ADEs were found in 20.4% of wild allele carriers (*p* = 0.009). Variant homozygous carriers of rs1042719 had a lower incidence rate of ADEs than did those with the G allele (*p* = 0.018).

**Table 2:** The occurrence of adverse drug events according to the grouped genotypes of *ADRB2*

Gene Polymorphisms	Minor Allele Frequency	Grouped Genotypes	Number of Patients	Patients with ADE (#)	Patients without ADE (#)	Odds Ratio (95% Confidence Interval)	*P*-value
rs1042713 A > G Arg16Gly	0.47	AA, AG	145	29	116	1	.167
		GG	41	4	37	0.432 (0.143–1.311)	
rs1042714 C > G Gln27Glu	0.10	CC	151	25	126	1	.461
		CG, GG	35	8	27	1.493 (0.608–3.666)	
rs1042717 G > A Leu84Leu	0.36	GG, GA	162	33	129	1	.009
		AA	24	0	24	0.843^a^ (0.787–0.903)	
rs1042718 C > A Arg175Arg	0.36	CC, CA	162	33	129	1	.009
		AA	24	0	24	0.843^a^ (0.787–0.903)	
rs1042719 G > C Gly351Gly	0.46	GG, GC	146	31	115	1	.018
		CC	40	2	38	0.195 (0.045–0.854)	

^a^Data were expressed as relative risks

*ADE* Adverse Drug Event

The maximum infusion rate of ritodrine (AOR 4.47, 95% CI 1.31–15.25) and the presence of *ADRB2* rs1042719 were both significant predictors of ADEs (Table 3). After adjusting for related covariates, carriers of the CC genotype in rs1042719 had approximately 80% lower incidence of ADEs than did those with other genotypes. The Hosmer–Lemeshow test showed that the fitness of the model was satisfactory (χ^2^ = 14.69, *p* = 0.07).

**Table 3:** Potential predictors of adverse drug events

Variables	Adjusted Odds Ratio	95% Confidence Interval		*P*-value
		Lower	Upper	
Age	.999	.900	1.124	.994
BMI	1.063	.944	1.198	.315
Maximum Infusion Rate (mg/min)	4.471	1.311	15.251	.017
ADRB2 rs1042719				
GG, GC	1			.043
CC	.215	0.048	.952	

## Discussion

The central findings of this paper are that the *ADRB2* gene polymorphism (rs1042719) may decrease ADEs induced by ritodrine and the maximum infusion rate of ritodrine can increase the incidence of ADEs.

The first issue to consider is the mechanism by which ritodrine—and β2-agonists in general—induces ADEs. Such ADEs depend on the specificity of β2-agonists to the myocardium or their selectivity to ADRB2. In the former case, stimulation of β1-adrenergic receptors (ADRB1) in the heart results increases cardiac output by increasing stroke volume and heart rate. In the latter case, ADEs are caused by the lack of uterine selectivity of β-agonists [9, 16]. Stimulation of ADRB2 in the vasculature causes relaxation, which leads to hypotension and further tachycardia [17].

However, previous studies have shown conflicting results regarding the mechanism by which ritodrine causes cardiac ADEs. Using animal models, Inoue et al. showed that ritodrine was 40-fold more selective to ADRB2 than to ADRB1 [18]. However, an early clinical study on ritodrine suggested that ritodrine in clinical doses for inhibiting premature labor may lose its β2 selectivity [19]. Furthermore, Inoue et al. also suggested that ritodrine was relatively more selective to the uterine compared to the atrium (43-fold greater) [18]. On the other hand, another study found that intravenous ritodrine infusion decreased vagal cardiac baroreflex sensitivity and vagal heart rate modulation, thereby leading to an increase in the mean heart rate in pregnant women [20]. As such, the debate involving the mechanisms underlying ritodrine-induced ADEs seems to need further relevant research.

As previously mentioned, it is worth assessing the possibility of ADRB2 stimulation leading to vasodilation, which could then cause hypotension and ultimately result in the advent of ADEs. In particular, the effect of gene polymorphisms should be addressed in this context. In this issue, previous studies have focused on a SNP with substitution of glycine (Gly) for arginine (Arg) at amino acid position 16 (rs1042713). Systemic infusions of selective β2-agonists have shown to result in greater vasodilation in Arg16 than Gly16 homozygotes [21, 22]. Our study suggested similar results, demonstrating that the wild type allele of rs1042713 was associated with an increased rate of ADEs, although statistical significance was not obtained.

**Fig. 1:**
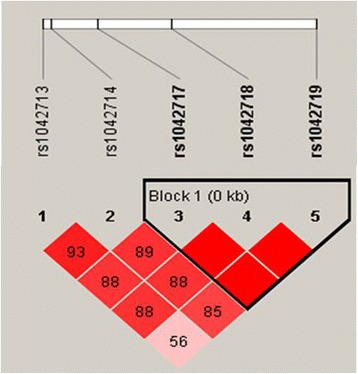
Linkage disequilibrium patterns and relative position of rs 1,042,717, rs1042718, and rs1042719 in study patients

Candidate SNPs that were utilized in this study were selected from both previous research and the Haploview Program. Litonjua et al. identified three major *ADRB2* gene polymorphisms that influence the ADRB2 response; Arg16Gly (rs1042713), Gln27Glu (rs1042714), and Thr164Ile (rs1800888); all three are missense mutations [13]. Among these, variants of rs180088 were not found in the Asian population [23], and thus only rs1042713 and rs1042714 were included in this study. In addition, candidate SNPs were selected through Haploview, first by filtering for those with minor allele frequencies (MAFs) of ≥20% in the Japanese and Han Chinese populations and second by constructing LD blocks (Fig. 1). The MAF criterion was adopted to ensure statistical power, since the inclusion of polymorphisms with lower MAFs could hinder statistical validity. In result, three additional SNPs (rs1042717, rs1042718, and rs1042719) were identified, and ultimately, a total of five SNPs were genotyped in this study.

Among the studied SNPs, rs1042717, rs1042718 and rs1042719 are synonymous polymorphisms, and these were significantly associated with ADE occurrence after ritodrine therapy in our population. While it was expected that synonymous SNPs would be insignificant, recent studies have suggested that they could affect molecular processes. Synonymous SNPs were shown to have a substantial effect on mRNA translation rates and gene expression, resulting in different protein isoforms and post-translational modifications [24, 25]. Therefore, it would be unwise to ignore the possible role of synonymous SNPs in our study as well.

In order to identify the characteristics involved in the occurrence of ADEs to ritodrine, multivariate logistic regression analysis was performed based on the univariate results (*p* < 0.1). We also included age and body mass index, which are strong confounders in clinical studies. In the final model, maximum infusion rate (reflecting the maximum dose) and genetic variation of rs1042719 were significantly related to ADE occurrence with ritodrine.

One of the predictors of ritodrine-induced ADEs, the infusion rate, is known to be significantly correlated with increased heart rate, possibly secondary to decreased baroreflex sensitivity [20]. In this study, ritodrine-induced ADEs were significantly correlated with the maximum infusion rate (mg/min) as a continuous variable (*p* = 0.006). Further analysis identified that patients who received ritodrine at an infusion rate of 0.15 mg/min or higher had a 6.9-fold increased risk of ADEs compared to those with an infusion rate < 0.15 mg/min. However, total amount of ritodrine was not significantly different between the two groups, although adverse event group (9894.9 ± 12,823.7 mg) showed higher dose than no adverse event group (7158.8 ± 10,385.6 mg) (*p* = 0.19). This was explained because the ritodrine administration was ceased or infusion rate was reduced when ADEs occurred.

On the other hand, the other predictor, namely the SNP rs1042719, has been seldom emphasized in previous literature. Perhaps the nature of rs1042719 as a synonymous polymorphism had caused such scarcity of relevant research; however, as mentioned before, there have been several papers hinting at the possible significance of synonymous polymorphisms [24, 25]. Furthermore, in our prior study on the efficacy of ADEs, rs1042719 had been once again identified as a significant factor, suggesting that this SNP might have a profound effect on the mechanisms of ritodrine. Therefore, it would be prudent to delve into the role of rs1042719, particularly in light of our results.

In univariate analysis, carriers of rs1042717 variants (rs1042718) had a significantly lower incidence rate of ADEs than did wild type carriers. However, this SNP was excluded in multivariate analysis, because there was no patient with variant type homozygote in the adverse event group. Therefore, the calculation of an odds ratio was unavailable.

## Conclusions

The rs1042719 polymorphism of the *ADRB2* gene and the maximum ritodrine infusion rate were associated with ritodrine-induced ADEs including tachycardia, tremor, palpitations, dyspnea, and pulmonary edema. The polymorphism of rs1042719 was also found to be a significant factor for time to delivery after ritodrine administration in our previous study; patients with CC genotype showed decreased efficacy [14]. Therefore, these results could help clinicians consider both efficacy and safety in pursuing personalized ritodrine therapy with genetic polymorphisms. However, this study was limited by its inclusion of a single center and the small sample size. Therefore, in the future, larger studies incorporating patients of different ethnicities would be needed to clarify the role of *ADRB2* gene polymorphisms in ADE occurrence.

## Methods

### Patients and data collection

This study is a prospective follow-up study which was conducted between January 2010 and February 2014, at Ewha Womans University Mokdong Hospital. The Ethics Committee and Institutional Review Board (IRB number: 217–1-26) approved this study. Data collection and patient inclusion adhered to the study protocol as described in the previous study [14].

Patients that met the following criteria were eligible for participation: preterm labor with intact membranes, gestational age of 20–36 weeks, ≥18 years of age, and uterine contractions with a frequency of 3 per 10 min with cervical change. Patients with the following high-risk conditions upon admission were excluded: placental abruption, fetal distress, severe spontaneous premature rupture of membranes, pre-eclampsia, and major vaginal bleeding. Patients with maternal conditions that precluded detection of outcomes, including hyperthyroidism, asthma, and cardiovascular disease, were also excluded. Patients in whom ritodrine was used to prevent uterine contractions during the Mcdonald operation or who were treated with tocolytics other than ritodrine were also excluded. Before participation, written informed consent was obtained from every patient.

Ritodrine (Lavopa®; JW Pharmaceutical, Seoul, Korea) was administered intravenously at an initial infusion rate of 0.05 mg/min. The infusion rate was increased by 0.05 mg/min every 10 min until the desirable uterine response was obtained. The intravenous treatment was discontinued during uterine quiescence. Patients who achieved uterine quiescence received maintenance therapy with an infusion of 0.05 mg/min for 12–48 h.

The collected patients’ information included maternal age, body weight, body mass index, gestational age, comorbidity, time of initiation and termination of ritodrine therapy, ritodrine dose, and type of adverse event. ADEs were defined as the presence of tachycardia, palpitations, dyspnea, tremor, or pulmonary edema, which was confirmed by a doctor as ritodrine-induced ADEs. The study population was divided into two groups based on the occurrence of ADEs: adverse event group and no adverse event group.

### Genotyping

Blood samples were collected for genotyping during the admission. Genomic DNA was extracted from ethylenediaminetetraacetic acid-blood samples using the QIAamp DNA Blood Mini Kit (QIAGEN GmbH, Hilden, Germany) according to manufacturer’s recommendations. Based on previous studies, two missense SNPs (rs1042713 and rs1042714) were selected for genotyping. In addition, genetic information of the *ADRB2* genes was incorporated into the Haploview Program for the selection of *ADRB2* SNPs with a MAF of ≥20% in the Japanese and Han Chinese populations. The linkage disequilibrium blocks were constructed following the D’-method in Haploview [26]. A total of 5 SNPs (rs1042713, rs1042714, rs1042717, rs1042718, and rs1042719) were genotyped in this study.

### Statistical analysis

In order to identify characteristics associated with ADEs, we performed univariate analysis using Fisher’s exact test for categorical data and t-test for continuous variables. A multivariable logistic regression model was used to identify independent predictors after adjusting other variables with a *p*-value <0.1 on univariate analysis in addition to the well-known confounders of age and body mass index. For the model’s goodness of fit, a Homer-Lomeshow test was performed. All statistical tests were conducted with a two-tailed alpha of 0.05. All analyses were performed using Statistical Package for Social Sciences Version 20.0 for Windows (SPSS 20.0 K, SPSS INC., Chicago, IL, USA).

## Abbreviations

ADE: Adverse drug event
ADRB1: β1-Adrenergic receptor
ADRB2: β2-Adrenergic receptor
cAMP: Cyclic adenosine monophosphate
LD: Linkage disequilibrium
MAF: Minor allele frequency
SNP: Single nucleotide polymorphism

## References

[1] WHO (1977). Recommended definitions, terminology and format for statistical tables related to the perinatal period and use of a new certificate for cause of perinatal deaths. Acta Obstet Gynecol Scand.

[2] Ventura SJ, Martin JA, Curtin SC, Mathews TJ (1998). Report of final natality statistics, 1996. Mon Vital Stat Rep.

[3] Goldenberg RL, Culhane JF, Iams JD, Romero R (2008). Epidemiology and causes of preterm birth. Lancet.

[4] Liu YL, Nwosu UC, Rice PJ (1998). Relaxation of isolated human myometrial muscle by beta2-adrenergic receptors but not beta1-adrenergic receptors. Am J Obstet Gynecol.

[5] Wray S (1993). Uterine contraction and physiological mechanisms of modulation. Am J Phys.

[6] de Heus R, Mol BW, Erwich JJ, van Geijn HP, Gyselaers WJ, Hanssens M (2009). Adverse drug reactions to tocolytic treatment for preterm labour: prospective cohort study. BMJ.

[7] Driul L, Londero AP, Adorati-Menegato A, Vogrig E, Bertozzi S, Fachechi G (2014). Therapy side-effects and predictive factors for preterm delivery in patients undergoing tocolysis with atosiban or ritodrine for threatened preterm labour. J Obstet Gynaecol.

[8] Neilson JP, West HM, Dowswell T (2014). Betamimetics for inhibiting preterm labour. Cochrane Database Syst Rev.

[9] Leineweber K, Heusch G (2009). Beta 1- and beta 2-adrenoceptor polymorphisms and cardiovascular diseases. Br J Pharmacol.

[10] Jacobson GA, Yee KC, Wood-Baker R, Walters EH (2015). SULT 1A3 single-nucleotide polymorphism and the single dose pharmacokinetics of inhaled salbutamol enantiomers: are some athletes at risk of higher urine levels?. Drug Test Anal.

[11] Ortega VE (2015). Predictive genetic profiles for beta-agonist therapy in asthma. A future under construction. Am J Respir Crit Care Med.

[12] Liggett SB (2002). Polymorphisms of the beta2-adrenergic receptor. N Engl J Med.

[13] Litonjua AA, Gong L, Duan QL, Shin J, Moore MJ, Weiss ST (2010). Very important pharmacogene summary ADRB2. Pharmacogenet Genomics.

[14] Park JY, Lee NR, Lee KE, Park S, Kim YJ, Gwak HS (2014). Effects of β2-adrenergic receptor gene polymorphisms on ritodrine therapy in pregnant women with preterm labor: prospective follow-up study. Int J Mol Sci.

[15] Hawkins GA, Weiss ST, Bleecker ER (2008). Clinical consequences of ADRbeta2 polymorphisms. Pharmacogenomics.

[16] Finley J, Katz M, Rojas-Perez M, Roberts JM, Creasy RK, Schiller NB (1984). Cardiovascular consequences of beta-agonist tocolysis: an echocardiographic study. Obstet Gynecol.

[17] Hadi HA, Abdulla AM, Fadel HE, Stefadouros MA, Metheny WP (1987). Cardiovascular effects of ritodrine tocolysis: a new noninvasive method to measure pulmonary capillary pressure during pregnancy. Obstet Gynecol.

[18] Inoue Y, Yoshizato T, Kawarabayashi T (2009). Investigation of beta(2)-adrenoceptor subtype selectivity and organ specificity for bedoradrine (KUR-1246), a novel tocolytic beta-adrenergic receptor stimulant. J Obstet Gynaecol Res.

[19] Vesalainen RK, Ekholm EM, Jartti TT, Tahvanainen KU, Kaila TJ, Erkkola RU (1999). Effects of tocolytic treatment with ritodrine on cardiovascular autonomic regulation. Br J Obstet Gynaecol.

[20] Bieniarz J, Ivankovich A, Scommegna A (1974). Cardiac output during ritodrine treatment in premature labor. Am J Obstet Gynecol.

[21] Gratze G, Fortin J, Labugger R, Binder A, Kotanko P, Timmermann B (1999). Beta-2 adrenergic receptor variants affect resting blood pressure and agonist-induced vasodilation in young adult Caucasians. Hypertension.

[22] Hoit BD, Suresh DP, Craft L, Walsh RA, Liggett SB (2000). Beta2-adrenergic receptor polymorphisms at amino acid 16 differentially influence agonist-stimulated blood pressure and peripheral blood flow in normal individuals. Am Heart J.

[23] Maxwell TJ, Ameyaw MM, Pritchard S, Thornton N, Folayan G, Githang'a J (2005). Beta-2 adrenergic receptor genotypes and haplotypes in different ethnic groups. Int J Mol Med.

[24] Pagani F, Baralle FE (2004). Genomic variants in exons and introns: identifying the splicing spoilers. Nat Rev Genet.

[25] Raponi M, Baralle D (2010). Alternative splicing: good and bad effects of translationally silent substitutions. FEBS J.

[26] Gabriel SB, Schaffner SF, Nguyen H, Moore JM, Roy J, Blumenstiel B (2002). The structure of haplotype blocks in the human genome. Science.

